# Little Italy: An Agent-Based Approach to the Estimation of Contact Patterns- Fitting Predicted Matrices to Serological Data

**DOI:** 10.1371/journal.pcbi.1001021

**Published:** 2010-12-02

**Authors:** Fabrizio Iozzi, Francesco Trusiano, Matteo Chinazzi, Francesco C. Billari, Emilio Zagheni, Stefano Merler, Marco Ajelli, Emanuele Del Fava, Piero Manfredi

**Affiliations:** 1 Department of Decision Sciences, Bocconi University, Milan, Italy; 2 Department of Computational Social Science, George Mason University, Fairfax, Virginia, United States of America; 3 Sant'Anna School of Advanced Studies, Pisa, Italy; 4 Dondena Centre for Research on Social Dynamics, Bocconi University, Milan, Italy; 5 Department of Demography, University of California, Berkeley, Berkeley, California, United States of America; 6 Predictive Models for Biomedicine & Environment, Bruno Kessler Foundation, Trento Povo, Italy; 7 Interuniversity Institute for Biostatistics and Statistical Bioinformatics, Hasselt University, Diepenbeek, Belgium; 8 Dipartimento di Statistica e Matematica Applicata all'Economia, Università di Pisa, Pisa, Italy; Imperial College London, United Kingdom

## Abstract

Knowledge of social contact patterns still represents the most critical step for understanding the spread of directly transmitted infections. Data on social contact patterns are, however, expensive to obtain. A major issue is then whether the simulation of synthetic societies might be helpful to reliably reconstruct such data. In this paper, we compute a variety of synthetic age-specific contact matrices through simulation of a simple individual-based model (IBM). The model is informed by Italian Time Use data and routine socio-demographic data (e.g., school and workplace attendance, household structure, etc.). The model is named “Little Italy” because each artificial agent is a clone of a real person. In other words, each agent's daily diary is the one observed in a corresponding real individual sampled in the Italian Time Use Survey. We also generated contact matrices from the socio-demographic model underlying the Italian IBM for pandemic prediction. These synthetic matrices are then validated against recently collected Italian serological data for Varicella (VZV) and ParvoVirus (B19). Their performance in fitting sero-profiles are compared with other matrices available for Italy, such as the Polymod matrix. Synthetic matrices show the same qualitative features of the ones estimated from sample surveys: for example, strong assortativeness and the presence of super- and sub-diagonal stripes related to contacts between parents and children. Once validated against serological data, Little Italy matrices fit worse than the Polymod one for VZV, but better than concurrent matrices for B19. This is the first occasion where synthetic contact matrices are systematically compared with real ones, and validated against epidemiological data. The results suggest that simple, carefully designed, synthetic matrices can provide a fruitful complementary approach to questionnaire-based matrices. The paper also supports the idea that, depending on the transmissibility level of the infection, either the number of different contacts, or repeated exposure, may be the key factor for transmission.

## Author Summary

Data on social contact patterns are fundamental to design adequate control policies for directly transmissible infectious diseases, ranging from a flu pandemic to tuberculosis, to recurrent epidemics of childhood diseases. Most countries in the world do not dispose of such data. We propose an approach to generate synthetic contact data by simulating an artificial society that integrates routinely available socio-demographic data, such as data on household composition or on school participation, with Time Use data, which are increasingly available. We then validate the ensuing simulated contact data against real epidemiological data for varicella and parvo-virus. The results suggest that the approach is potentially a very fruitful one, and provide some insights on the biology of transmission of close-contact infectious diseases.

## Introduction

A century after the first contributions giving birth to mathematical epidemiology, and after 20 years of fast growth since the first public health oriented contributions [1]–[3], infectious diseases modeling has recently received a further dramatic impulse from pandemics threats. The Bio-terrorism and SARS first, the fear of a potentially devastating pandemic of avian flu then, and finally the recent pandemic of A/H1N1 influenza, have all fostered the development of more and more detailed predictive tools. These range from traditional models to network analysis, to highly detailed, large scale, individual-based models (IBM) [4]–[17]. IBM are highly flexible tools for policy makers as they allow to define intervention measures at the finest possible levels (e.g., the contact network of single individuals during a specific activity). For the first time, a pandemic model on a continental scale has been proposed [17].

A critical aspect common to all such models, is the parameterization of social contact patterns, i.e. how people socially mix with each other [18]. Social contact patterns are the key factors underlying the transmission dynamics of directly transmitted close-contacts infectious diseases [18]. Different models, independently of their level of complexity or geographical scale, are sensitive to the parameterization of social contact patterns.

In a relatively simple case, where individuals are stratified by age only, contact patterns are represented in the form of contact matrices whose entries represent the average number of contacts that individuals in age group *i* have with individuals in age group *j*, per unit of time. Until recently, contact patterns were estimated “indirectly” by calibrating suitably restricted contact matrices using observed epidemiological data, such as serological or case notifications data. The two major examples of this indirect approach are the Who-Acquires-Infection-From-Whom (WAIFW) matrix [3], and the proportionate/preferred mixing approach [19]. Such approaches have important restrictions: in a population divided in *n* age groups, a contact matrix contains *n*x*n = n^2^* unknown entries. Therefore, in order to estimate the *n^2^* parameters from the *n* data points (e.g., serological data) some simplifying assumptions about the structure of the matrix are needed. In addition, indirect approaches can only estimate adequate contacts or transmission rates, i.e. composite parameters given by the product between a contact rate and the corresponding risk of infection per contact.

Recently, important progress has been made in this area through direct collection of contact data by means of sample surveys [20]–[25]. The direct approach is based on appropriate definitions of an “at risk event” (e.g., a face-to-face conversation). Survey respondents are then asked to record in a diary relevant characteristics (e.g., age) of all the individuals they had contact with during a randomly assigned day, or other factors such as the location where the contact occurred (e.g., home, school, public transportation). Standardized international survey data on social contact patterns in 8 European countries are currently available [24]. In addition, contact matrices, and “time in contact” matrices, have been estimated from secondary data sources such as transportation surveys [26] or time use data [27], which are increasingly available. In the case of time use data, the underlying hypothesis is that the amount of time people spend doing the same activity in the same place is relevant for the transmission of the disease.

A drawback of time use data is that they usually do not give direct information about the number of social contacts of respondents, or the time they spent in contacts. They only give “marginal” information on the time individuals allocated to the various daily activities [27]. Therefore, these data need to be augmented with other data and/or assumptions to produce reliable estimates of contact matrices [27]. A way to supplement time use data relies on socio-demographic sources (e.g., routine or census data) which provide information on the size and distribution of the “arenas” (e.g., school, workplaces, households) where contacts take place. For example, for school contacts we often know the average class size and the average pupils-teacher ratio for all compulsory grades. As for contacts within the household, we have information on household size and composition. For most other activities, however, there is little information. Assumptions, e.g. independency, are therefore necessary to give some coarse ideas of contact patterns [27]. However, this approach ignores the structure of the social networks where contacts are formed. A promising approach is then to reconstruct such networks by the simulation of appropriate artificial social networks. A first example is the social network generated by the “Portland” synthetic population [26]. In that case, “contact” and “time in contact” matrices by age are by-products of the social dynamics of the Portland model. These matrices have the standard expected features: population contacts cluster around children and adult, children interact most frequently with other children close to their own age, etc. However, such matrices were neither compared with other contact matrices, nor validated against empirical epidemiological data. Thus, no actual evaluation of their “goodness” in explaining transmission of infections is available.

In this paper, we follow the same line and aim to reconstruct contact and time-in-contacts matrices by simulating a suitable “minimalistic” socio-demographic individual-based model for Italy. The model is parameterized by integrating time use data from the Italian time use survey [28] and other official socio-demographic data [29]–[30]. In the model, each artificial agent is a “clone” of a real individual, i.e. there is a one-to-one correspondence between the diary of each “artificial” agent and the one of a corresponding “real” survey participant. Since the sample is representative of the Italian population, but the size of the model population is comparable to that of a small Italian city, we named the model “Little Italy”. From this point of view, our model resembles the Portland model [6], and the Eemnes (a small Dutch city) model [31]. In the Little Italy world, agents “physically” displace during the day in order to attend their various daily activities in the corresponding location. In these locations, agents “contact” other agents. We defined a contact as “having shared the same physical environment” (i.e. house, the same class at school, the same bus) during a given time slot.

With our approach we generate three different types of contact matrices, possibly informative of distinct aspects of the biology of transmission: (a) a matrix describing the time spent in contact (Type 1) [27], (b) a matrix counting the number of repetition of contact episodes (Type 2), and (c) a matrix counting contacts as the average number of different persons contacted, i.e. the number of different social partnerships, (Type 3) as in [24].

In addition, we extracted an adequate [19] contact matrix from the socio-demographic model underlying the Italian IBM for pandemic prediction and mitigation [15], that we named “Big-Italy”. The synthetic contact matrices computed by simulation of Little and Big-Italy are tested against recently collected Italian serological data on Varicella and ParvoVirus (B19). Their performances are compared with other contact matrices available for Italy, i.e. the “Polymod” and “time use” matrices.

## Materials and Methods

### Italian time use and routine data

The Italian Time Use (TU) survey was carried out by the Italian National Statistical Agency between 2002 and 2003 [28], with a sample of 55,773 individuals, grouped into 21,075 households. Respondents, with the exception of children less than 3 years old, were asked to fill in a questionnaire with a diary of the activities done during a randomly selected day. To take into account the differences between workdays and week-ends, the sample was divided into three groups. One group was asked to fill the diary on a given workday (18,085 diaries collected), one on a Saturday (16,828) and one on a Sunday (16,293).

A 24-hour day, starting from 4am, is divided into 144 time slots of 10 minutes each, called “ticks”. For each tick, the respondent's diary records the type of location where the person was, and the type of activity done. Due to privacy issues, records always refer to types of places and types of activities, instead of exact places and exact activities. Types of places and types of activity are given unique codes (i.e. 1 for home, 2 for office, etc. for locations; 1 for working, 2 for caring children, etc. for activities). However, these codes are identical for every individual. Therefore, if at the same chronological time two people are both working, each one in his/her own office, we have two records with the same codes, but this does not imply they are in the same office doing the same thing. This has some drawbacks. First, there is never any clue about the purpose of the undertaken activity. For example, if in a certain tick someone reports being on the public transportation network, there is no indication about the reasons for being there. For instance, it could be for going from home to office, or bringing children, if any, to school and then going to work, or anything else. The same applies for places, with a single remarkable exception: if at any time two individuals report that they are at home, and we know from other data that they both belong to the same household, we can infer that they are in the same place. This is the only case in which the partial information given by respondents can be correctly augmented.

Finally, routine socio-demographic data on a) family size and composition [28]; b) firms size by number of employees [29]; and c) school class size for any school grade [30], were used to inform our model.

### Building Little Italy

To create an artificial society that matches the one that is revealed by the Time Use survey, some assumptions were made. Unlike other approaches (e.g., the Portland model), whose aim is to create artificial societies that are as close as possible to a real population, we opted for an artificial society based on a “minimally” complex set of rules, that is nonetheless representative of the Italian population. This seems to be a useful departure point: by considering a simple spatial structure and a minimal set of activities/locations (school and work, the household, and “other”, non-school, non-household contacts), which are those considered fundamental in basic epidemiological explanations, we avoid the need to include several extra-assumptions for model parameterization. Further activities and locations can nonetheless be easily included.

Let us list the assumptions adopted. First, we restricted our model to individuals followed over an average workday. This choice sets Little Italy's population to 18,085 (artificial) individuals. We chose to ignore week-end days because the groups of respondents to the surveys are different and therefore some additional assumptions would be necessary to link workdays and week-end days agendas.

During the day, agents move to and from different places. Most of the time, respondents reported to be at home, in the office or at school. For the rest, they either declared to be in more specific places (e.g., bakery, park, etc.) or that they were moving from one place to another (e.g., on foot, by car or by bus). We chose a square grid as Little Italy's “environment”, with grid's size 150×150, in order to allocate families in single cells representing houses, leaving appropriate space for schools and workplaces. Each square in the grid is identified by a pair of coordinates. We allocated one house for each household on a random cell on the grid. House cells can contain at most 5 families.

In order to host all students aged 3–18, and Little Italy's only university, we allocated schools at random on the grid.

The setting up of workplaces required a few more assumptions, since respondents only reported that they were at work during some ticks but gave no information about either the size of the company they were working for, or the number of colleagues (and in many situations, like, for instance, bus drivers, workers “share the environment” with people that are not necessarily colleagues). Therefore, we drew samples of firms from the workforce size distribution of Italian firms in cities having population size comparable to Little Italy, i.e. 10,001–20,000 inhabitants [29]. This yielded a number of alternative configurations for the number of firms, and for their sizes, which are representative of the real variability observed in small Italian towns having the size of Little Italy. We finally put each firm on a single random cell and assigned each worker to a firm.

Two aspects of the previous process are worth mentioning. First, each agent declares how much time she/he spends going, say, from home to office by car. This time is a proxy for the distance from home to office which must be respected all over Little Italy. It is not possible, for example, that agent A takes 1 time slot (10 minutes) to move by 20 cells on the grid while agent B covers the same distance in 6 time slots (1 hour) if they both declare using the car. This would mean that A's car moves 6 times faster than B's one, which is possible, but unlikely. We proceeded as follows. After workers are assigned to firms, a random re-assignment of houses is performed: two households exchange their houses if, in the new configuration, the actual distances between offices and houses are closer to the ones that can be inferred from their diaries. A large number of exchanges is carried out until the error cases are a negligible fraction of total workers.

Second, there are workers who declared not having a single workplace, like, for examples, a plumber. For them, we decided to set their moving workplace at random. Each time they are about to go somewhere, the simulation chooses a random square on the grid as their next workplace. Commercial places are created on the grid and their workers are assigned to them. Students are assigned to classes in schools, according to routine data [30], which prescribe an average number of students per classroom on a regional basis. These figures are very close to the observed ones [28]. The related numbers (averaged over Italy) are: 25 for individuals less than 2 years old; 23 for individuals from 3 to 5; 18 for kids from 6 to 10; 21, from 11 to 13, and 22 for teenagers (14–18). Higher education is represented by a university with about 700 students.

To run Little Italy, at every tick each agent must be put somewhere on the grid. This requires each agent's list of activities to be put in a one-to-one correspondence with a pair of coordinates. This, in turn, requires a detailed modelling of the agents movements over Little Italy. Details are reported in Text S1.

### Simulation of Little Italy

Little Italy was coded in Java, using Repast 3 libraries [32]. We first drew a large number of alternative configurations in the number of firms and their sizes. From this initial set we discarded those configurations which resulted to be inconsistent with Little Italy. From the consistent set, we selected at random a subset of 100 “worlds”. For each of these worlds, we ran 100 single-day (i.e. each one lasting 144 ticks) simulations. Results obtained from multi-day simulations are not considered because of the limited variability: most agents in Little Italy have small stochastic components in their daily life, the only random elements being the displacement of their house, their office and the paths they follow during the day.

### Computation of Little Italy contact matrices

To keep track of contacts between agents, a definition of contact was necessary. The adopted “marker” of contact was “having shared the same physical environment with someone else” (i.e. house, the same class at school, the same bus) during a given tick. This corresponds to a form of localized random mixing. Assume, for instance, that during a given tick there are 20 pupils aged 7 and one teacher aged 44 in a class-room. Based on our definition each pupil has 19 contacts with people of the same age, and one contact with adult people (aged 44), while the teacher has 20 contacts with 7 years old people.

By aggregating across time ticks, matrices reporting the total number of contacts between each pair of ages were computed for the following activity/locations: household, school, work, transport, other activities. Then, by summing through activities we computed overall (i.e. including contacts through all locations) contact matrices by age, whose elements *K_ij_* represent the total number of contacts between individual in age-groups *i* and *j*. In fact, three different types of contact matrices were computed (we call them Type 1,2,3 matrices). They represent, respectively, the time in contact, the number of episodes of contact, and the number of social partnerships. As an illustrative example, assume that two agents of age *i* and *j*, respectively, share the same square on the grid for 6 ticks, then they are elsewhere for some time, and finally they “meet” again for two further ticks. These agents will contribute to the element *K_ij_* of the Type 1 matrix with 8 units of “time in contact”. On the other hand they contribute to element *K_ij_* of the Type 2 matrix with only 2 contact episodes. Finally, using the definition of contact commonly adopted in surveys [21]–[24], our two individuals contribute to the element *K_ij_* of the Type 3 matrix with only 1 unit of contacts. Note that all Types of matrices are symmetric by definition (“If individual A has shared a given location with B, then also B must have shared the same location with A”).

From total matrices *K_ij_* and the number *n_i_* of individuals in each age group, we computed standard mean contact matrices, i.e. matrices whose entries are the mean numbers *m_ij_* of contacts with individuals having age *j* per individual having age *i*, using the (symmetric) relation *K_ij_* = *m_ij_n_i_* = *m_ji_n_j_* = *K_ji_*.

Since Little Italy matrices do not offer information on contacts for the age group 0–2 years (it is not included in the Italian Time Use Survey), we integrated our matrices using Polymod data from that age group. These computations were applied to each “world”, and then the average of the ensuing matrices was taken.

We note that the different types of contact matrices considered correspond to different views of the contact process, perhaps useful to capture different aspects of the biology of transmission. Type 1 matrix might be relevant for infections for which the time of exposure matters (for instance, for those infections with low transmissibility rates, where the probability of transmission cumulates over time). Type 3 matrix implies that what really matters is the number of social partnerships, independently of the number of repetition of contact episodes and of the time spent together [24]. Type 2 lies in between, i.e. the transmission depends on repetition of contacts but not necessarily on their duration.

### The Big-Italy matrix

The Big-Italy matrix was extracted from the IBM used to simulate the spread and control of an influenza pandemic in Italy [15]. In this model, differently from Little Italy, each Italian individual is explicitly represented by a model agent. This agent is characterized by age, household membership, and school/workplace membership. The ensuing synthetic population has been obtained by using official socio-demographic data only. In other words, differently from the Little Italy population, it does not include time use data. Since the Big-Italy agents do not physically displace among the different types of locations, only one “general” contact matrix could be computed from the simulation of Big-Italy, by counting the number of contacts among the model agents and then weighting each contact by considering the location where the contact took place (namely households, schools, workplaces, or general community). Details on the computation of the Big-Italy matrix are given in Text S2.

### Other contact matrices

We compared the performances of the Little and Big Italy matrices with two other contact matrices available for Italy: a) the overall (i.e. including all reported contacts) Polymod matrix based on survey data collected in eight European countries [24]; b) the Time Use matrix obtained with the methodology described in [27]. The matrix in (b) relies on the same Time Use Survey as Little Italy, but does not use additional socio-demographic data.

### Serological data

Recently collected Italian serological data (age range 0–79 years, sample size = 2,517) on varicella-zoster-virus (VZV) [33], and ParvoVirus B19 [34] were used. For these infections no mass vaccination programme is in place in Italy, so that their observed immunity profiles may be assumed to represent pre-vaccination equilibrium.

### Fitting serological data, transmission rates, R_0_

Fitting contact matrices to serological data was performed using a standard approach [22], [27], i.e. by plugging mean contact matrices into a simple age-structured SIR transmission model at its endemic equilibrium. The equilibrium force of infection (FOI, the per-capita probability to acquire the infection per unit of time) is therefore constant in each age group *i* defining the contact matrix, with the form: 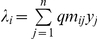, where 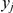 denotes the infective prevalence at equilibrium in each age group (defined as the ratios between the number 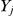 of infective people in group *j* at equilibrium, and the corresponding population size 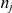), and *q* is a single age-independent transmission parameter. By formally solving the model at equilibrium, and letting *D* and 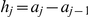 denote, respectively, the average duration of the infective period and the size of the *j*-th age group, one gets 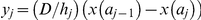, where 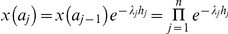 is the susceptible fraction at exact age 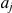. The equilibrium FOI in each age group is then determined by solving the system of *n* nonlinear equations 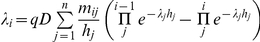. Once the equilibrium FOI is available, for any given *q*, the predicted immunity profile at equilibrium *z(a)* at any given age *a* is computed as 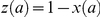. Finally, the fitting was carried out by maximizing the likelihood of the transmission parameter *q* in the explanation of the observed age-specific proportions of people immune to VZV and B19 in Italy.

The *one-q strategy* is a clear way to compare contact patterns, since it implies that the infection process only mirrors contact patterns rescaled by a constant representing infection transmissibility. Since the different contact matrices considered have different scales, the corresponding *q'*s have different units. For example *q* represents a transmission rate per single tick of time for Little Italy Type 1, while it is measured per single episode of contact for Type 2, etc. The difference in units makes the *q*s of little direct comparability. For this reason, the comparison of the performances of the various matrices in explaining serological data is not based on the actual *q* estimates, but only on goodness of fit measures.

Given the estimate of the transmission parameter *q*, we can compute the “next generation matrices”, *NG_ij_ = qm_ij_*, from which the corresponding Basic Reproduction Number *R_0_* can be obtained [35]. In these cases, *R_0_* is a measure of the potential of invasion of an infection with transmissibility *q* in a community whose contact patterns are summarized by the contact matrix of elements *m_ij_*.

### Non parametric fit

In order to achieve a high degree of explanatory power of the data to be used as a benchmark of the goodness of fit of the various matrices, we also considered a flexible non parametric model, given by a constrained monotonically increasing P-splines model [36] (details in Text S3).

### Assortativeness measures

We measure assortativeness in the various matrices considered using two different indices. The first one is the *Q* index [37], defined as 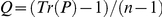 where *P* = [*p_ij_*] is the matrix whose elements *p_ij_* represent the fractions of total contacts of age group i with age group j: 
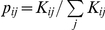
 and 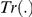 denotes the Trace of the matrix. The *Q* index ranges between zero, corresponding to proportionate mixing, and one, under full diagonal dominance, i.e. fully assortative mixing. Therefore *Q* represents a measure of departure from proportionate mixing for groups defined on a qualitative scale. The second measure is the dissimilarity-type index 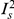 [38], defined as the mean square deviation from perfect assortativeness of the contact distribution 
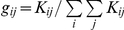
, normalized by its value under homogeneous mixing. This index is a normalized measure of disassortativeness, ranging between 0, when assortativeness is perfect, and 1, when mixing is homogeneous. For symmetric contact distributions, 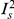 is related to the correlation coefficients *ρ_XY_* of the contact distribution as: 
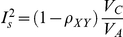
, where 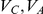 respectively denote the variances of the marginal distribution of contacts with age, and of the age distribution of the population.

## Results

### Little Italy contact matrices

**Figure 1:**
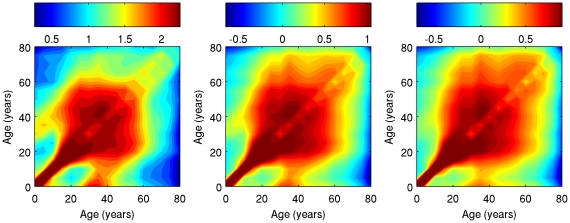
Contour plot of Little Italy contact matrices (contacts in log scale). Type 1 (left), Type 2 (center), Type 3 (right). X-axis = age of the contactors, Y-axis = age of his/her contacts.

**Figure 2:**
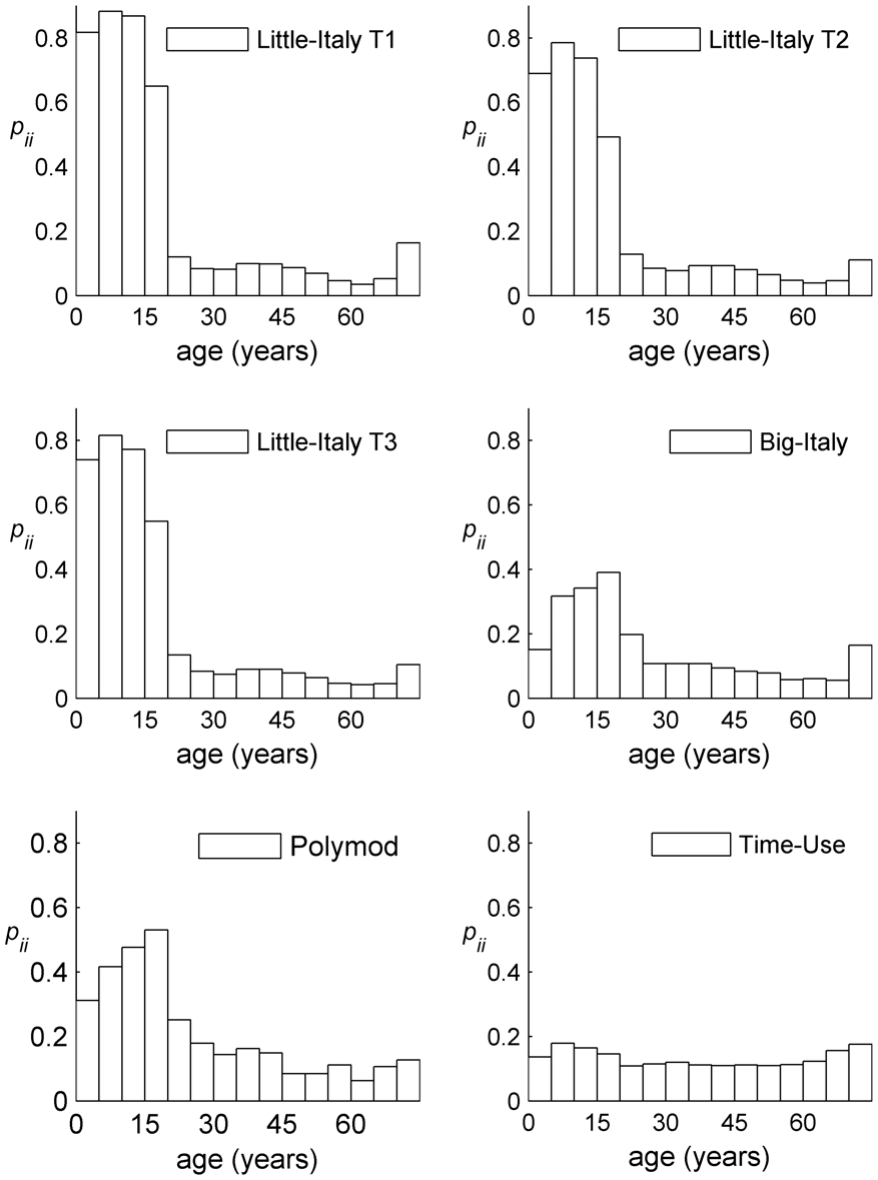
Proportions of contacts with individuals of the same age. Proportions *p_ii_* of contacts with individuals of the same age group, for each age group, in the six contact matrices considered.

Contour plots of Type 1, 2, and 3 average contact matrices based on 5-years age groups (0–4, 5–9, etc) are reported (Fig. 1). The three matrices are very assortative, i.e. the majority of contacts are on the main diagonal, meaning that individuals tend to have contacts with people of the same age. Assortativeness, however, varies significantly across age: it is very pronounced in children, according to the stylized fact that most contacts occur with school classmates, which essentially are of the same age. In particular, the three Little Italy matrices are largely more assortative than all the other matrices considered: for individuals who are less than 15 years old, the proportion of contacts that individuals in each age group have with other individuals in the same age group ranges between 75 and 85% in the three Little Italy matrices, whereas it ranges between 25 and 55% in the Polymod matrix, even less in the Big Italy matrix (Fig. 2). This larger assortativeness of Little Italy matrices is confirmed by the measures 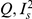 (Table 1).

**Table 1:** Assortativeness measures for the various contact matrices.

	*Q*	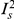
Little Italy Type1	0.225	0.316
Little Italy Type 2	0.184	0.412
Little Italy Type 3	0.195	0.428
Big Italy	0.094	0.661
Polymod	0.157	0.632
Time-Use	0.070	0.569

Values of selected measures of assortativeness for the various contact matrices considered.

With regards to contacts between parents and their children, well evidenced in [24], [27], these clearly appear in Type 1 matrix (the stripes above and below the main diagonal), whereas they are less sharply defined in Type 2 and 3 matrices. This is explained by the fact that Type 1 matrix takes into account the long time spent by children at home (in most cases with at least one parent) whereas Type 2 and 3 do not. Compared to the Polymod matrix, in all Little Italy matrices household contacts are quantitatively less important because of the stronger assortativeness, which dominates non diagonal contacts. In addition, the lack of appropriate information in the time use diaries probably prevented several contacts between parents and children to be accounted for, thus leading to under estimation. Overall, we can say that Little Italy matrices are dominated by school contacts as a consequence of the assumptions made. The activity-specific matrices used to compute the Type 1 matrix are reported in Text S4.

### Fitting contact matrices to serological data

**Figure 3:**
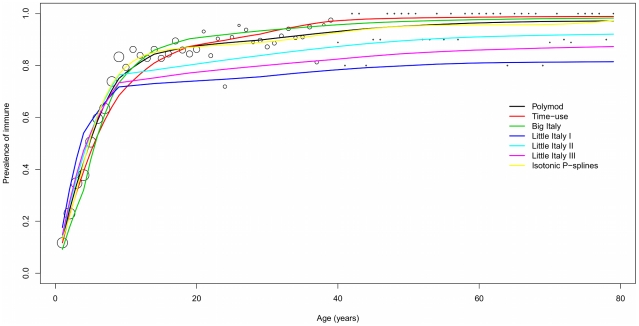
Graphic view of the fit to VZV data. Fit to Italian serological data for VZV by an SIR model based on the various contact matrices considered: observed vs predicted immunity profiles to VZV, by age. Dots size proportional to sample frequency of serological data.

**Figure 4:**
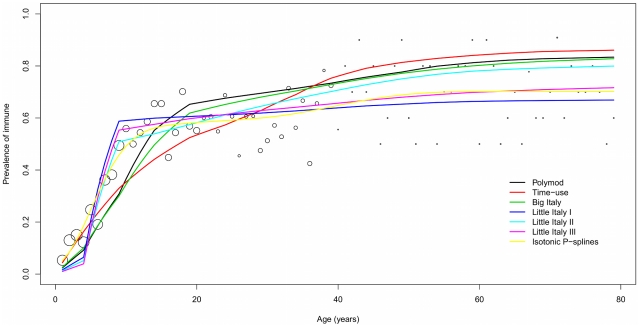
Graphic view of the fit to B19 data. Fit to Italian serological data for B19 by an SIR model based on the various contact matrices considered: observed vs predicted immunity profiles to B19, by age. Dots size proportional to sample frequency of serological data.

Table 2 reports the main output of the fit (optimal *q* estimates, deviance and Akaike Information Criterion, and the corresponding estimates of the Basic Reproduction Number *R_0_*) to VZV and B19 data, for all the matrices considered. Results from the non-parametric model are also included. Graphic comparisons between observed and predicted sero-profiles by age are displayed in Fig. 3 and Fig. 4.

**Table 2:** Results of fit to serological data.

		*q*	*Deviance*	*AIC*	*R_0_*
**VZV**	Little Italy Type1	0.051 (0.047,0.055)	276.42 (1 df)	447.61	3.14
	Little Italy Type 2	1.35 (1.29, 1.42)	111.37 (1 df)	282.57	4.94
	Little Italy Type 3	1.42 (1.35,1.51)	190.15 (1 df)	361.34	3.43
	Big Italy	12.35 (11.67,13.09)	101.11 (1 df)	272.30	4.80
	Polymod	11.37 (10.80, 11.99)	67.34 (1 df)	238.53	4.77
	Time-Use	4.28 (4.09,4.47)	114.32 (1 df)	285.51	4.11
	Non-parametric		64.30 (4.92 df)	243.33	
**B19**	Little Italy Type1	0.029 (0.028, 0.030)	135.61 (1 df)	402.11	1.72
	Little Italy Type 2	0.73 (0.71, 0.75)	157.24 (1 df)	423.74	2.67
	Little Italy Type 3	0.82 (0.80, 0.84)	159.90 (1 df)	426.39	1.98
	Big Italy	5.39 (5.20, 5.60)	195.99 (1 df)	462.48	2.10
	Polymod	5.26 (5.06, 5.48)	202.91 (1 df)	469.41	2.21
	Time-Use	2.23 (2.16, 2.30)	195.60 (1 df)	462.09	2.14
	Non-parametric		81.23 (3.95 df)	353.63	

Results of the fit to Italian serological data for VZV and B19 by an SIR model based on the various contact matrices considered: *q* estimates and related 95% confidence intervals (column 3), deviance and related number of degrees of freedom (df, column 4), Akaike information criterion (AIC, column 5), R_0_ estimates (column 6). Deviance and AIC also reported for the non-parametric model.

For VZV, the Polymod matrix provides the best fit. The Big-Italy and the Little Italy Type 2 matrices perform better than the Time Use matrix but substantially worse than the Polymod matrix, whereas the Little Italy Type 1 and 3 matrices fit poorly. Note that the non-parametric model performs slightly better than the Polymod matrix in terms of deviance, but worse in terms of AIC, due to its larger parameterization. This suggests that the Polymod matrix definitely represents an excellent “explanans” for VZV transmission. Disregarding the Little Italy Type 1 and 3 matrices, which poorly fit, the ensuing values for 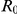 are in good mutual agreement (ranging between 4 and 5), and higher than the *R_0_* estimates reported for Italy in [33]. We also note that both the poorly fitting Little Italy Type 1 and 3 matrices lead to much smaller *R_0_* values. This follows from the limited ability of these matrices to capture contact patterns relevant for VZV. As a result, we observe a compensation through anomalously small values of the infectivity parameter *q*.

Things are different for B19. The Type 1 matrix provides the best fit, and overall the three Little Italy matrices perform better than the other matrices. It is however to be acknowledged that the fit remains far from the one provided by the non-parametric model, suggesting that there is still room for large improvements in the explanation. In particular, the Big-Italy and the Time Use matrices, though clearly less performant than the Little Italy matrices, are not worse than the Polymod matrix. The ensuing values for R_0_ range between 1.6 and 2.6. An explanation of the differences in the fit of B19 and VZV is not easy since we do not dispose of tools to globally compare the differences between two arbitrary contact matrices. Assortativeness measures provide however some clue. The three Little Italy matrices predict a very steep immunity profile at low ages, which however suddenly flattens to a plateau later on. This sudden change in regime, which is a pattern known to occur in presence of strong assortativeness, allows the Little Italy matrices to better explain the B19 data, which show a sharp plateauing (though with large randomness). On the other hand, this behavior prevents the Little -Italy matrices to capture the observed VZV profile.

Finally, given that the large-scale (transport and shopping malls) contacts of the Little Italy model required several assumptions to be parameterized, it was important to check the influence of these activities/locations on the result of the fit. We therefore fitted the Little Italy matrices without taking into account such activities, i.e. relying only on households and school/workplaces contacts. The results in the fit of B19 by Little Italy Type 1 and 3 matrices are reported (Table 3). In the situation where the Little Italy model performs better, i.e. the fit of B19 by the Type 1 matrix, the exclusion of transportation and shopping malls worsens the goodness-of-fit very little, indicating that these components only marginally affect the structure of the matrices.

**Table 3:** Results of fit to serological data after removal of “large scale” contacts.

		*q*	*Deviance*	*AIC*	*R_0_*
Little Italy Type 1	All contacts	0.0293 (0.0284, 0.0301)	135.61 (1 df)	402.11	1.716
	Without transportation	0.0293 (0.0284, 0.0301)	135.65 (1 df)	402.14	1.713
	Without transportation & malls	0.0294 (0.0286, 0.0303)	138.36 (1 df)	404.85	1.661
Little Italy Type 3	All contacts	0.818 (0.796, 0.842)	159.90 (1 df)	426.39	1.982
	Without transportation	0.826 (0.804, 0.850)	160.70 (1 df)	427.2	1.96
	Without transportation & malls	0.867 (0.844, 0.893)	200.11 (1 df)	466.61	1.639

Fit to B19 data by Little Italy Type 1 and Type 3 matrices: comparison between the case where all contacts are considered vs the cases where: a) contacts on transportations are excluded, and b) also contacts on shopping malls are excluded. Figures for the “All contacts” case are the same as in Tab. 2.

## Discussion

Substantial improvements have been achieved in recent times in our knowledge of social contact patterns [20]–[27], which are thought to be a key factor underlying the transmission dynamics of close-contact infections. In this paper, we have investigated the potentialities of IBM as a tool for the generation of contact data, with two distinct approaches. The first approach is a novel one, based on a simple socio-demographic IBM (“Little Italy”) strictly integrating time use and routine socio-demographic data. As for the second, we have extracted the contact matrix by age (“Big-Italy”) implicit in the socio-demographic model underlying the Italian IBM for pandemic prediction and control. Both models are based on the same routine socio-demographic data, but the “Little Italy” model also considers the agents' daily allocation of time through Time Use data. The Little Italy approach allows for the computation of different types of contact matrices, labelled Type 1,2,3, reflecting respectively (a) the average time in contact, (b) the average number of repetition of contacts, (c) the average number of different persons contacted.

The ensuing contact matrices by age were fitted, on the basis of simple transmission models, to Italian serological data for VZV and B19. Goodness-of-fit comparisons with other available contact matrices, such as the questionnaire-based Polymod matrix and the Time-use matrix, were also made. The main results show that for VZV the best fit is provided by the Polymod matrix, which performs excellently, and much better than artificial matrices. However, for B19, all Little Italy matrices fit the data quite well, and better than available concurrent matrices, including the Polymod one.

This paper represents, as far as the authors know, the first comparison on real epidemiological data of bottom-up approaches to the generation of contact data, with the approaches based on direct contacts estimation, such as the Polymod study. Our results on VZV provide further evidence on the merits of the Polymod study, which represents a great advancement in our understanding of contact patterns. However, the better fit to B19 provided by artificial matrices compared to questionnaire-based matrices, is indicative of the difficulty to find “universal” contact patterns that can explain in a satisfactory way many different infections. Therefore, though artificial matrices can not surrogate observed ones, they can certainly represent valuable tools to assist mathematical modellers in the formulation of alternative assumptions.

An important related question is why different infections are better explained by different types of contact matrices. May this be due to the characteristics of the contacts which matter to various infectious diseases? The traditional WAIFW [3] and proportionate mixing [19] approaches, which were strongly constrained by data availability, considered the various diseases separately, as if they were outcomes of fully independent processes. Recent approaches [20]– have promoted the different idea that for a large family of infections there might be a unique “core” of observable social contact patterns, mediated through a unique, or a few, infection-specific transmission parameters. These new approaches raise a number of questions: first of all, whether the transmission biology of different infections could selectively exploit different types of contact patterns. Though this is still unclear, there is evidence that the infection-specific hit probabilities per single viral or bacterial unit occupy a wide range. This would suggest that for some infections, such as measles, even very short single episodes of contacts might be sufficient for transmission. Therefore, it is likely that most adequate contacts are usually “wasted”. On the other hand, there might be infections (e.g., bacterial ones) characterized by a very low hit probability, for which many repetitions of contact episodes, or long exposure times, might be necessary for successful transmission. Our results, i.e. the fact that for a mildly transmissible infection such as B19, the best fit is obtained using a matrix counting time spent in contacts, as opposite to VZV, where the best fit follows from a matrix only counting encounters with different individuals, irrespective of time of exposure, might support this idea.

With regards to model parameterization, the Little Italy model uses real data to parameterize the small scale components (household sizes, schools, workplaces) of the contact network. On the other hand, assumptions were necessary to parameterize the large scale components of the network, e.g. travel and shopping malls patterns. Nonetheless, we could at least make such patterns fully consistent with the general design of the Little Italy model, i.e. the daily time spent on travelling, or in supermarkets, by each Little Italy agent, correctly matches, based on an optimization procedure, the time spent on travelling by a corresponding real agent. In order to appreciate the potential impact on data fitting of the ad-hoc assumptions on travel patterns, we also fitted the model by excluding contacts on transports and shopping mall, showing that in the most significant cases the results were essentially unaffected. This suggests that the “empirically robust” component of the model is sufficient for the main target of the paper, i.e. the generation of contact data. Obviously, given the lack of appropriate epidemiological data to validate travel assumptions, the possibility to use Little Italy for further investigations beyond those presented here, i.e. for example epidemic prediction and information of measures targeting social distance, certainly requires caution. Future work will be devoted to the analysis of the model robustness to the assumptions on its large scale components.

Given the simplicity of the adopted definition of contact, the current model cannot reproduce, unless resorting to further data and hypotheses, the richness of data obtained by Polymod survey, where further noteworthy information such as the intimacy and frequencies of contacts, were collected. This is clearly a shortcoming since these types of contacts are arguably important for most respiratory infections [24].

However the current Little Italy model can potentially be used to answer several important questions. For example, the model can be expanded to describe contacts in a rural-urban environment, given the representativeness of Italian Time Use data for rural and urban populations. Moreover, longer time simulations could address how contacts cumulate (a) during periods of time having a length comparable to the infectivity period, (b) between work-days and week-end days [39]. We indeed recall that, although in this paper we considered work-days only, the Italian Time Use data actually include three distinct samples, one for working days, the other two for Saturdays and Sundays. This provides information on how time spent in the various activities/locations cumulates through the different parts of the week. Obviously, studies of contacts accumulation are difficult, as they are necessarily conditional on the specific assumptions made on the larger-scale topology of the Little Italy network, e.g. contacts on transportations, shopping malls, and so on. Nonetheless, in recent times, the first empirical evidence on this issue has become available [25], and may provide a useful starting point for comparison of contact accumulation in different social settings.

Further, this paper would like to reinforce the perspective that contact data and time-use data provide useful complementary information. On the data-gathering side, major gains could be achieved by combining the two approaches. This could be achieved, for example, by supplementing time-use surveys with a few questions about people “contacted” (for example those with whom a conversation was held) during any given activity or time slot. This would provide data that consistently incorporate the relationship between time of exposure and contacts. With regards to studies of transmission, it would be important to better understand how to integrate the two types of data, for example by comparing time use data and Polymod data on durations of contacts.

A final point regards the information embedded in age specific serological data, which are the base for infection control strategies. As clear for example for VZV [33], these data show a fast monotonic increase during school ages, say up to age 10–15, then the trend becomes flat, or slightly increasing with age, but with large randomness. This suggests that these data have little discriminating power about infection patterns at higher ages, which are critically important when control measures are in place. Therefore, it would be important to improve our understanding of infection patterns among adults, for example by grounding stochastic models of age mixing against simulation derived matrices (and related seroprofiles by age). On a related topic, in our models we are still relying on the assumption of monotonic seroprofiles. This assumption follows from postulating an infection which (a) is at steady state, and (b) decouples from the underlying dynamics of the population. If these hypotheses are not met, seroprofiles can become non monotonic. Recent work [40]–[41] has aimed at considering infection dynamics in non-steady populations, or non steady contact networks. This work has suggested the importance of population structures in shaping contact patterns, and therefore the intrinsic instability of contact matrices over time. Time is ripe for bringing such non stationary approaches also in epidemiological data analyses.

## Supporting Information

Text S1 Little Italy details.
(0.12 MB PDF)

Text S2 Big Italy details.
(0.19 MB PDF)

Text S3 Description of the nonparametric model used to ground fitting results.
(0.02 MB PDF)

Text S4 Little Italy Type 1 activity-specific matrices.
(0.41 MB PDF)
