# Supplementary material for: Little Italy: An Agent-Based Approach to the Estimation of Contact Patterns- Fitting Predicted Matrices to Serological Data
Source: PLoS Comput Biol. 2010 Dec 2;6(12):e1001021. doi: 10.1371/journal.pcbi.1001021 (PMC2996317; doi:10.1371/journal.pcbi.1001021)
Supplement: Text S1 — Little Italy details. (0.12 MB PDF) [file pcbi.1001021.s001.pdf]

Little Italy: an Agent-Based Approach to the Estimation of Contact Patterns Fitting Predicted Matrices to Serological Data

Fabrizio Iozzi, Francesco Trusiano, Matteo Chinazzi,
Francesco C. Billari, Emilio Zagheni, Stefano Merler,
Marco Ajelli, Emanuele Del Fava, Piero Manfredi

Supporting Text 1

Little Italy Environment

Little Italy environment is composed by two 2-dimensional grids: the space of the city (or city-space) and the space of transports (or transport-space). The city-space contains houses, workplaces, schools and is where agents spend most of their time. The transport-space lies underneath the city-space and is where public and private transport means can be found.

The Space of the City

The city-space grid is a 150x150 square which comprises 22500 cells. Each cell is characterized by several variables:

- a set of Cartesian coordinates and a cell ID that uniquely identify each cell;
- a type variable that indicates the cell type (e.g. house, workplace, school, etc..);
- a capacity variable that defines the maximum capacity of each cell (used for classrooms and workplaces' sizes);
- an array variable that records the list of occupiers of the cell.

When the grid is initialized, status, capacity and array variables have empty values for all cells. The values of each of these variables will then be initialized when a specific "world-state" is loaded. Each "world-state" determines the distribution and characteristics of workplaces in the city-space, setting their type (either commercial workplaces or not-commercial), number and size (i.e. number of employees per local unit). The main difference between commercial and not-commercial workplace cells is that the latter ones can contain only agents who work in those cells; while the former ones, which represent places like bakeries, restaurants, shops, etc.., can also be visited by external random agents (i.e. customers). House cells, instead, are configured to contain at most

5 families, regardless of the world-state used. Houses and workplaces are then randomly distributed on the grid. To create classroom cells (i.e. schools and the university), some of the empty cells left on the city-space are chosen at random and converted into classroom cells. The overall number of created classrooms is computed considering the number of students observed for each school grade: the total capacity of all classroom cells for a particular schooling year must be equal to the number of individuals attending that year of schooling. For instance, if there are 56 children attending the second grade, we need to have a total school capacity of 56 when summing up all classroom cells capacities for second grade students. Classroom cells sizes are fixed using the average number of students per class as explained in the article.

The Space of Transports

The transport-space grid is a 10x10 square. Each transport-space cell is split into two distinct cell-objects:

- a private transport-cell object, which will contain the agents using private transport means;
- a public transport-cell object, which will contain the individuals using public transport.

The transport-space is used by the agents to move from one city-space cell to another and contains the entire Little Italy transportation network (roads, railways, underground lines, etc..). When agents are changing location, they do so by moving from the city-space down to the transport-space, going to their destination and finally popping back up in the city-space. A convenient metaphor of the interaction between the two spaces is a two levels city, with the underground (the transport-space) under the earth surface (the city-space). When an agent moves from a location to another one using public transportation, he goes down to the underground – where he interacts with other public transportation users - and at the end of his journey he comes back up to the street level. To cover the same area, the transport-space has fewer but bigger cells than the city-space. This means that for each transport-space cell there is a 15x15 square of city-space cells above it. This way of modeling the transportation network allows us to aggregate moving agents whose departure or destination city-space cells are close enough (i.e. within a 15x15 city-space square).

The Agents

Characteristics and Number of Agents

Each agent has 11 internal states:

1. ID: a counter identifying the agent;
2. FamId: a counter identifying the family the agent belongs to;
3. Age: the age of the individual;
4. Sex: the sex of the individual;

5. SchoolAge: the class attended by the agent if he is a student;
6. Workplace: the type of workplace where the agent works, which depends on the kind of economic activity performed (commercial or not-commercial);
7. SchoolDistance: the distance of a student from his school as reported in the time-use survey;
8. WorkDistance: the distance of a worker from his workplace as reported in the time-use survey;
9. House-cell: the ID and the coordinates of the agent's house in Little Italy;
10. Workplace-cell: the ID and the coordinates of the agent's workplace in Little Italy;
11. School-cell: the ID and the coordinates of the agent's school in Little Italy.

Notice that the school-age of an individual is defined as the age that one should have given that he is attending a certain class, regardless of one demographic age. For instance, if a person is 17 years old but claims to be attending the second year of high school, his school-age will be 15, not 17. Therefore, the school-age of an agent is not necessarily equal to his demographic age. With the school-age variable we solved the problem of putting in the same classroom people attending the same year of schooling but having different ages. This is crucial for correctly taking into account students' social interactions.
In Little Italy, there are five different classes of workers:

1. home workers;
2. commercial workers;
3. not-commercial workers;
4. school workers;
5. no-fixed-place workers, i.e. workers without a fixed workplace (e.g. a plumber).

This distinction is used by the model to determine the exact type of cell where an agent is supposed to be working. That is, a not-commercial worker must work in a not-commercial workplace cell; while, a school worker must be employed by a school and so on.

Placement of the Agents on the Space of the City

After that house, workplace and classroom cells have been created on the city-space, agents are randomly assigned to them, checking that:

- agents belonging to the same family are kept together in the same house cell;
- commercial workers are assigned to commercial workplace cells;
- not-commercial workers are assigned to not-commercial workplace cells;

- school workers (lecturers and school staff) are equally distributed among classroom cells; and
- each classroom cell is filled with students having the same school-age.

Then, we adjust agents positions on the city-space using WorkDistance and SchoolDistance variables so that agents' travel-times in Little Italy are as close as possible to the ones declared by each individual in the time-use survey. The correspondence between virtual and real travel-times is crucial in the construction of agents' daily routes, given that the time each individual spends traveling in our virtual world is basically equal to the one he spends in the real world. Hence, the need to carefully check agents' positions on the city-space, since their virtual positions will also determine their virtual travel-times. Little Italy randomly chooses a pair of agents at the time and checks if they are better off switching their positions on the city-space, evaluating the discrepancies between virtual and real distances before and after the switch. To do so, it uses each agent preferred house-workplace virtual distance which is created from the answers provided by the individuals in the time-use survey. The preferred distances of the two agents are compared first with their current house-workplace virtual distances, and then with the house-workplace distances in the case in which the agents are actually switched. Consequently, the algorithm checks if both agents are made better off switching, i.e. if the preferred-distance is closer to the switched-distance rather than to the current-distance. Let us denote with D_i^P the preferred-distance of agent i, with D_i^C the current-distance and with D_i^S the switched-distance. Then, two agents are switched of their positions if and only if:

$$|D_1^P - D_1^C| > |D_1^P - D_1^S| \text{ and } |D_2^P - D_2^C| > |D_2^P - D_2^S|$$

This entire process is repeated 10^6 times for each simulation, each time choosing a different pair of agents.

The Daily Travel Routes of the Agents

In the model, the house of an individual, his workplace and his school (or university) are called *fixed-places*, since those are all the places that always remain constant for each agent. This means that each agent always has the same house cell, the same workplace cell (this is obviously true only for the agents that have a sedentary work, not for the no-place workers) and the same school cell. Agent's daily travel route is a schedule which determines at each tick of the simulation where each agent has to be found in Little Italy virtual environment. It describes agent's movements on the city and transport grids. Daily routes creation starts with Little Italy reading the agent's diary (obtained from the time-use survey) looking for the first fixed-place that appears in it. Because the diary stores a list of types of place visited by each agent at a particular time during the day, it is sufficient for the algorithm to find types of places that correspond to each agent's house, school or workplace. Furthermore, each type of place is associated with an integer ranging from 1 to 144, corresponding to the time of the day when the agent was in each of the types of places listed in the diary. Indeed, the day is divided in 144 ticks; hence, one tick every ten minutes. The first tick refers to the location visited between 04:00–04:10 AM, while the last tick, 144, refers to 03:50–04:00 AM.

Our parsing algorithm reads each individual diary and looks for types of places corresponding to the codes 1 (house), 2 (workplace) and 3 (school). Then, it takes the first fixed-place appearing in the diary as the starting type of place for the agent at hand. Consequently, the model retrieves the coordinates of the first fixed-place from the agent's internal states. Afterwards, Little Italy repeats this operation iteratively, completing the travel-route of each agent with all the other fixed places visited after the first one. However, a special case has to be mentioned here. Some agents have an agenda where there are no fixed places that match the definition given above. This may happen, for instance, when an agent stays at a friend's house for the night. In such special cases, the algorithm searches for these different types of places instead of the usual ones, and uses those cells as agents starting points. Besides such fixed-places, the algorithm traces also the movements of the agents in other locations (e.g. shopping malls). In such cases, when the location may be different and not fixed over time, the algorithm chooses commercial places that will fit in the actual agenda of each individual. For example, if a person, while going home from office, declared to travel for 3 ticks, to stop somewhere to go shopping for a tick and then go home in the last 2 ticks, the algorithm firstly determines a random place where the agent could be after 3 ticks, then finds a suitable commercial place in the neighborhood, makes the agent go in the commercial place (and allow him to contact other agents who happen to be there at that moment), and then moves the agent back home. In principle, the previous algorithm could lead to an overcrowding into commercial places which cannot be acceptable. After that each agent has a travel-route on the city-space, the model proceeds by completing its route also on the transport-space. Little Italy assigns to each agent the transport-space cells on which the subject will pass through while he is on the move. To do so, an algorithm first counts the number of ticks that each agent spends traveling, then it calculates agent starting and ending coordinates on the transport-space accordingly. Those are the coordinates of the cells on the underground transport-space that lies exactly underneath the departure and arrival cells of the fixed-places located in the city-space. Once the program knows each agent starting and ending coordinates in the transport-space, it calculates which cells each agent will pass through and at what time during the simulated day (i.e. at what tick). As a consequence, we can map agents movements on our artificial transport network knowing exactly agents positions at each tick of the simulation.
