# Supplementary material for: Little Italy: An Agent-Based Approach to the Estimation of Contact Patterns- Fitting Predicted Matrices to Serological Data
Source: PLoS Comput Biol. 2010 Dec 2;6(12):e1001021. doi: 10.1371/journal.pcbi.1001021 (PMC2996317; doi:10.1371/journal.pcbi.1001021)
Supplement: Text S2 — Big Italy details. (0.19 MB PDF) [file pcbi.1001021.s002.pdf]

# Little-Italy: an agent-based approach to the estimation of contact patterns. Fitting predicted matrices to serological data.

Fabrizio Iozzi, Francesco Trusiano, Matteo Chinazzi, Francesco Billari, Emilio Zagheni, Stefano Merler, Marco Ajelli, Emanuele Del Fava, Piero Manfredi

Supporting Text S2

## Contents

**1 Sociodemographic structure of the simulated population** **2**

**2 Computing the Big–Italy Matrix** **3**

**Bibliography** **5**

# 1 Sociodemographic structure of the simulated population

Simulated agents, representing "real" individuals, were randomly grouped into households to match the 2001 census data on age structure (Italian Institute of Statistics, *XIV Censimento generale della popolazione e delle abitazioni*, 2001; available in Italian at url `http://dawinci.istat.it/MD/`) and data from a specific 2003 survey on household size and composition (Italian Institute of Statistics, *Strutture familiari e opinioni su famiglia e figli*, 2003; available in Italian at url `http://www.istat.it/dati/catalogo/20060621_03/`). Nine different types of households were considered in the model (e.g., singles or couples, with or without children, with or without additional members, adults living together). Individuals were co–located in households according to specific data on household type and size, and on the age of the household head. This procedure allows the simulated population to match marginal distributions of age structure of the population, household size and type, and to maintain realistic generational gaps within households (i.e., by avoiding random assignation of ages to households members). The frequency distribution of household sizes for the different types is shown in Fig. S1a, together with the frequency distribution of household types. Fig. S1b shows a comparison between the age structure of the simulated population and the 2001 census data.

At the time of the census, the Italian population was composed by 20,559,595 workers, 11,360,556 students and 25,084,274 unemployed or retired individuals. Children and young adults were assigned to one out of six levels of school (i.e., from day care center to university) on the basis of age and specific data on school attendance by age (Italian Ministry of University and Research, *La scuola in cifre* and *L'università in cifre*, 2005; both available in Italian at url `http://statistica.miur.it/ustat/documenti/pub2005/index.asp`). This allows considering the actual mix of student ages within schools. Attendance to school varies widely with age: 14% in day care centers, 90% in kindergartens, approximately 100% in primary and middle schools, 82% in high schools, 31% in university. We used specific data on employment rate by age to assign an employment to individuals aged more than 15 years. This ensures to account for the observed proportion of unemployed and retired individuals by age. Each worker was randomly assigned to one out of seven employment categories, defined on the basis of the number of employees in the workplace, in such a way to fit the available data on workplace size (Italian Institute of Statistics, *VIII Censimento generale dell'industria e dei servizi*, 2001; available in Italian at url `http://dwcis.istat.it/cis/index.htm`). Fig. S1c shows the comparison between simulated and observed size of the workplaces. Finally, teachers and school employees were also considered in the model by assigning a fraction of adult individuals to the simulated schools. The fraction of workers employed as teachers (or school employees) and the their age distribution were determined by using the data collected by the Italian Ministry of University and Research.

Further details on the sociodemographic structure of the simulated population can be found in Ciofi degli Atti et al. [1].

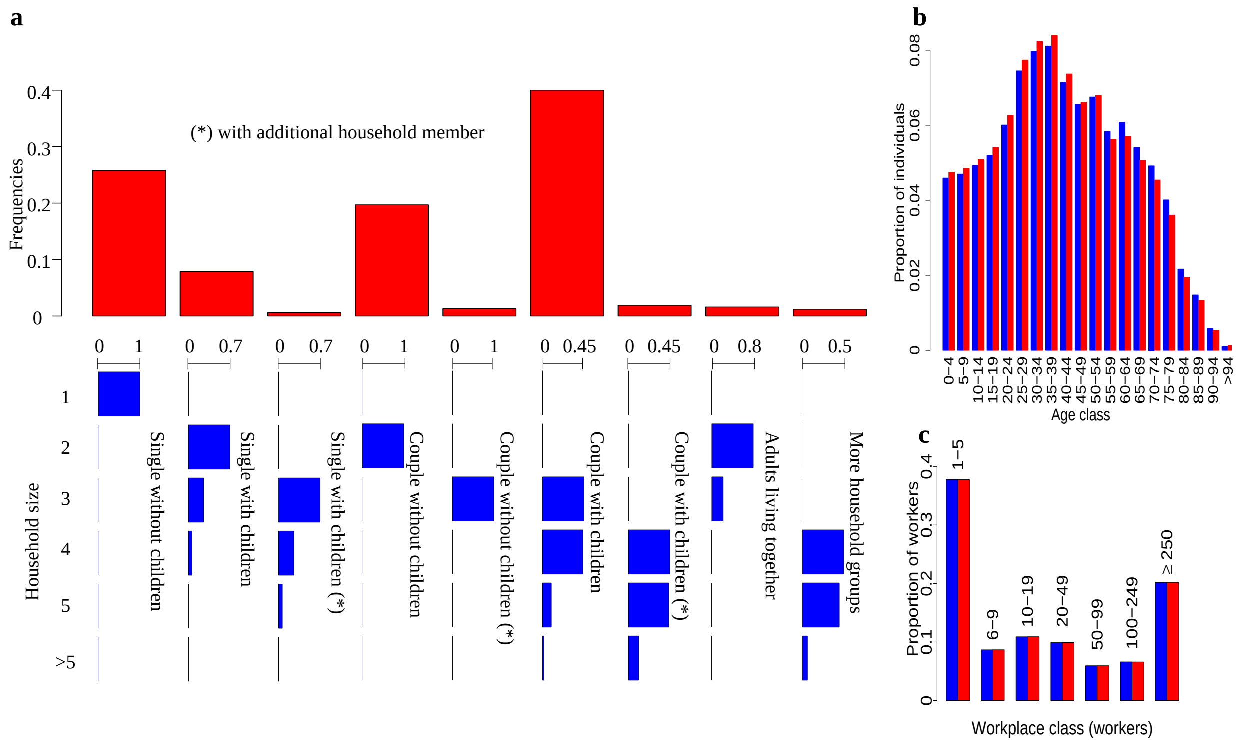


Figure S1: **a** Frequency distribution of the different household types considered in the model (red). Frequency distributions of household size for the different household types (blue). **b** Age distribution as resulting from census data (blue) and model simulations (red). **c** Workers by class of workplace size as resulting from industry census data (blue) and model simulations (red).

# 2 Computing the Big–Italy Matrix

The Big–Italy matrix $B$, whose elements $i, j$ represent the average number of contacts between individuals in age groups $i$ and $j$, is defined as a linear combination of the matrices accounting for contacts within household members (matrix $H$), within school/workplace colleagues (matrix $P$) and in the general community (matrix $R$).

The $i, j^{\text{th}}$ element of matrix $H$ (denoted by $H_{ij}$) represents the average number of individual of age $j$ contacted by an individual of age $i$ among the members of her/his household. Specifically, this matrix was computed by using the following procedure. For each individual $k$ of age $i$ living in household $h_k$ (whose size is denoted by $\bar{h}_k$), the household contacts with individuals of age $j$ were defined as the set of individuals of age $j$ living in $h_k$. These sets of contacts were determined directly by analyzing the structure of the simulated population. Therefore, $H_{ij}$ was estimated by averaging over individuals of class $i$, i.e.,

$$H_{ij} = \frac{1}{n_i} \sum_{\substack{1 \le k \le n_i \\ \bar{h}_k > 1}} (h_k^{(j)} - \delta_{ij})$$

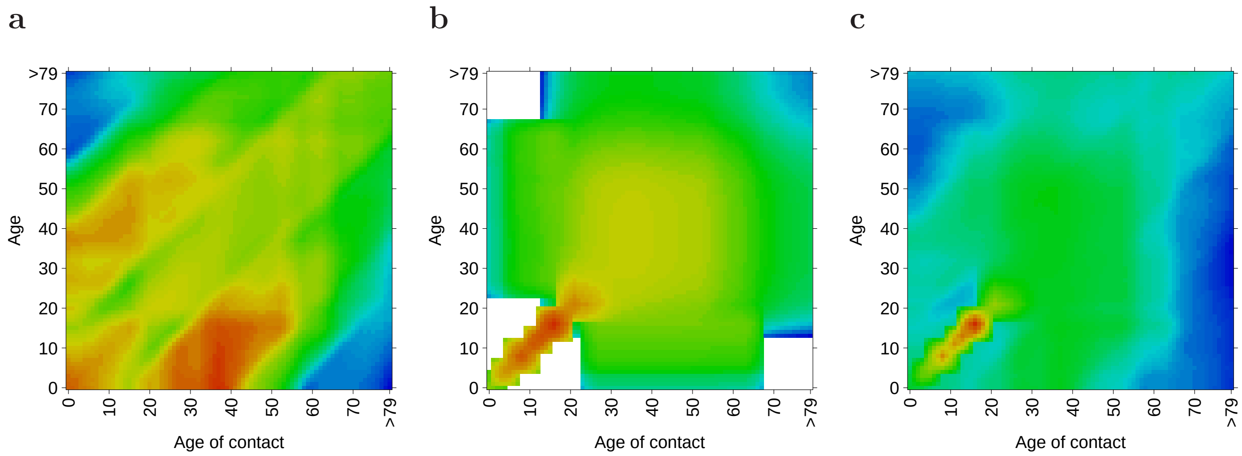


Figure S2: **a** Matrix $H$ (accounting for household contacts). Colors range from dark blue (small number of contacts) to red (large number of contacts). **b** Matrix $P$ (accounting for school and workplace contacts). Colors range from dark blue (small number of contacts) to red (large number of contacts); white regions stand for no mixing between age classes. **c** The Big–Italy matrix. Colors range from dark blue (small number of contacts) to red (large number of contacts).

where

- $n_i$ is the number of individuals of age $i$;
- $h_k^{(j)}$ is the number of individuals of age $j$ living in $h_k$ (i.e., the household where individual $k$ lives);
- $\delta_{ij}$ is the Kronecker delta function.

Matrix $H$ is shown in Fig. S2a.

The same procedure was used for computing matrix $P$ (whose $i, j^{\text{th}}$ element is denoted by $P_{ij}$), which accounts for school/workplace contacts. For each individual $k$ of age $i$ and attending school, or working in workplace, $p_k$ (whose size is denoted by $\bar{p}_k$), the school/workplace contacts with individuals of age $j$ were defined as the set of individuals of age $j$ attending $p_k$. Unemployed and retired individuals were thus not considered in this computation. $P_{ij}$ was computed as

$$P_{ij} = \frac{1}{n_i} \sum_{\substack{1 \leq k \leq n_i \\ \bar{p}_k > 1}} (p_k^{(j)} - \delta_{ij})$$

where

- $n_i$ is the number of individuals of age $i$;
- $p_k^{(j)}$ is the number of individuals of age $j$ attending place $p_k$;

- $\delta_{ij}$ is the Kronecker delta function.

Matrix $P$ is shown in Fig. S2b.

For contacts in the general community, homogeneous mixing among individuals was assumed. Therefore, the columns of matrix $R$ are proportional to the number of individuals by age.

In conclusion, the Big–Italy matrix $B$ (whose $i, j^{\text{th}}$ element is denoted by $B_{ij}$) is defined as the following linear combination of matrices $H$, $P$ and $R$:

$$B_{ij} = \varrho_1 \frac{H_{ij}}{\sum_{i=0}^{\Omega}\sum_{j=0}^{\Omega} H_{ij}} + \varrho_2 \frac{P_{ij}}{\sum_{i=0}^{\Omega}\sum_{j=0}^{\Omega} P_{ij}} + \varrho_3 \frac{R_{ij}}{\sum_{i=0}^{\Omega}\sum_{j=0}^{\Omega} R_{ij}}$$

where:

- $\Omega$ is the maximum age of the population (if a population divided into age classes is considered, $\Omega$ represents the number of age classes plus one);
- $\varrho_i$, for $i = 1, 2, 3$, are the linear combination coefficients.

The coefficients $\varrho_i$ are specific of a given disease. In fact, different kinds of contact can occur (e.g., speech or skin-to-skin contacts) in different environments and their relevance in the transmission depends on the pathogen responsible for the disease.

No specific information on the fraction of Varicella and Parvovirus B19 infections occurring in the three contexts (households, schools/workplaces and general community) is available. Therefore, the Big–Italy matrix was parameterized by considering values commonly used in influenza models, namely $\varrho_1 = 0.3$, $\varrho_2 = 0.37$, $\varrho_3 = 0.33$ [2, 3]. The Big–Italy matrix is shown in Fig. S2c.

# References

[1] M. L. Ciofi degli Atti, S. Merler, C. Rizzo, M. Ajelli, M. Massari, P. Manfredi, C. Furlanello, G. Scalia Tomba, and M. Iannelli. Mitigation measures for pandemic influenza in Italy: an individual based model considering different scenarios. *PLoS ONE*, 3(3):e1790, 2008.

[2] N. M. Ferguson, D. A. Cummings, C. Fraser, J. C. Cajka, and P. C. Cooley. Strategies for mitigating an influenza pandemic. *Nature*, 442(7101):448–452, 2006.

[3] S. Merler and M. Ajelli. The role of population heterogeneity and human mobility in the spread of pandemic influenza. *Proc R Soc B*, 277(1681):557–565, 2010.
