# Supplementary material for: Little Italy: An Agent-Based Approach to the Estimation of Contact Patterns- Fitting Predicted Matrices to Serological Data
Source: PLoS Comput Biol. 2010 Dec 2;6(12):e1001021. doi: 10.1371/journal.pcbi.1001021 (PMC2996317; doi:10.1371/journal.pcbi.1001021)
Supplement: Text S3 — Description of the nonparametric model used to ground fitting results. (0.02 MB PDF) [file pcbi.1001021.s003.pdf]

Little-Italy: an agent-based approach to the estimation of contact patterns. Fitting predicted matrices to serological data.

Fabrizio Iozzi, Francesco Trusiano, Matteo Chinazzi, Francesco Billari, Emilio Zagheni, Stefano Merler, Marco Ajelli, Emanuele Del Fava, Piero Manfredi

Supporting Text S3. Description of the nonparametric model used to ground fitting results

We use a monotonic increasing P-splines model (Eilers and Marx, 1996). P-splines consist in a least squares regression with an excessive number of univariate B-splines and an additional discrete penalty to correct for overfitting.

Given an interval $(k_{\min}, k_{\max})$ with a grid of q points k_i (called interior knots), a B-spline of degree q consists of q+1 polynomial components of degree q joined smoothly at the knots, and taking positive values over each component interval (k_{i-1}, k_i) and a value of zero outside the boundaries. To use B-splines in a nonparametric regression, we construct a B-splines basis, given by a family of r overlapping B-splines. The B-splines basis plays the same role as the predictor matrix in a classical regression model.

In order to select the appropriate number r of B-splines, we consider a very large number of B-splines, with a smoothness penalty consisting of the integrated squared second-order derivative of the fitted curve, in order to correct for overfitting. Finally, to obtain an monotonic (model (Bollaerts *et al*., 2006), we add a discrete penalty on n-th order differences, reflecting the hypothesized nonparametric functional form.

Let $\hat{y}(\alpha)_i$ be the fitted values depending on a set of coefficients α for the B-splines basis to be estimated. In particular λ represents the smoothness parameter (the smaller λ, the higher the degree of smoothness) and $\Delta^2\alpha_j$ the second-order differences that have the purpose to penalise the overfitting. Let moreover $w(\alpha)_j$ be the asymmetric weight, equal to 1 in case the n-th order differences $\Delta^n\alpha_j$ are negative (in case of monotonic increasing models). The score function is given by:

$$S = \sum_{i=1}^{m}(y_i - \hat{y}(\alpha)_i)^2 + \lambda\sum_{j=3}^{r}(\Delta^2\alpha_j) + \kappa\sum_{j=n+1}^{r} w(\alpha)_j(\Delta^n\alpha_j)^2$$

where the first term represents the mean square error of the spline regression, the second term represents the penalizing factor for overfitting, and the third one, depending on the n-th order differences, the penalizing factor for having the hypothesized (nonparametric) monotonic functional form.

Setting $\kappa = 10^6$, we use a Newton-Raphson algorithm to estimate the coefficients α of the model, while the optimal smoothness parameter λ can be chosen from a grid of values using methods like generalized cross-validation or the AIC.

References

Eilers, P. H. C., & Marx, B. D. (1996). Flexible smoothing using B-splines and penalized likelihood (with comments and rejoinder). *Statistical Science,* 11, 89–121.

Bollaerts, K., Eilers, P.H.C., and van Mechelen, I. (2006). Simple and multiple P-splines regression with shape constraints. *British Journal of Mathematical and*

Statistical Psychology,59, 451-469.
