# Supplementary material for: Little Italy: An Agent-Based Approach to the Estimation of Contact Patterns- Fitting Predicted Matrices to Serological Data
Source: PLoS Comput Biol. 2010 Dec 2;6(12):e1001021. doi: 10.1371/journal.pcbi.1001021 (PMC2996317; doi:10.1371/journal.pcbi.1001021)
Supplement: Text S4 — Little Italy Type 1 activity-specific matrices. (0.41 MB PDF) [file pcbi.1001021.s004.pdf]

Little-Italy: an agent-based approach to the estimation of contact patterns. Fitting predicted matrices to serological data.

Fabrizio Iozzi, Francesco Trusiano, Matteo Chinazzi, Francesco Billari, Emilio Zagheni, Stefano Merler, Marco Ajelli, Emanuele Del Fava, Piero Manfredi

Supporting Text S4. Little Italy activity-specific Type 1 matrices.

- We report contour plots of Little Italy Type 1 activity-specific matrices. These matrices are computed for the five Little-Italy principal activities:
  - Household
  - School
  - Work
  - “Other” activities (termed “commercial workplaces”)
  - Transport

- The matrices entries represent total time spent in contacts between individuals aged *i* and *j* for each single year of age. These matrices are by definition symmetric. The total contacts Little Italy Type 1 matrix K_{ij} used in the paper is obtained by summing the five activity specific matrices.

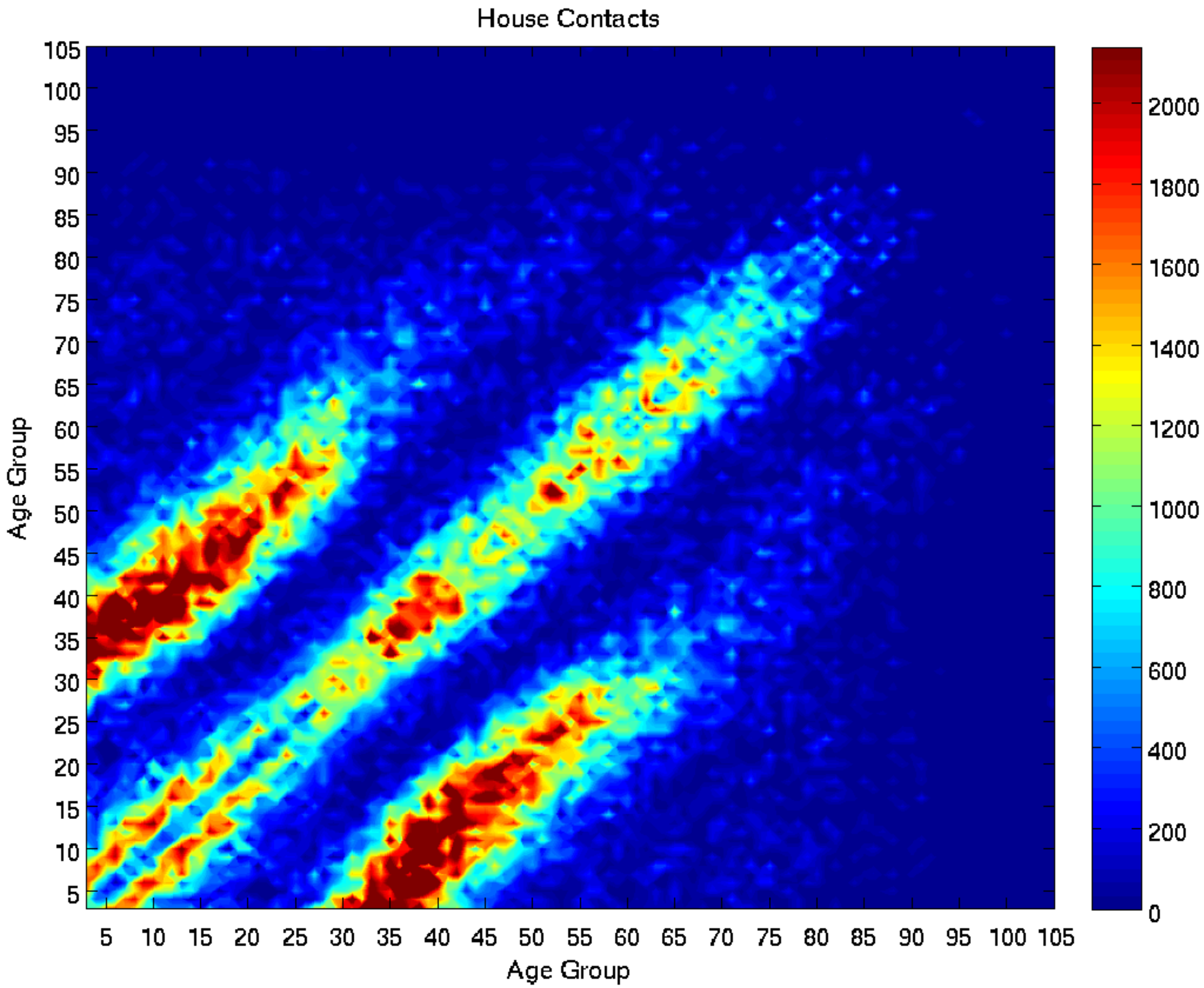
House Contacts
Age Group
Age Group
105
100
95
90
85
80
75
70
65
60
55
50
45
40
35
30
25
20
15
10
5
5 10 15 20 25 30 35 40 45 50 55 60 65 70 75 80 85 90 95 100 105
2000
1800
1600
1400
1200
1000
800
600
400
200
0

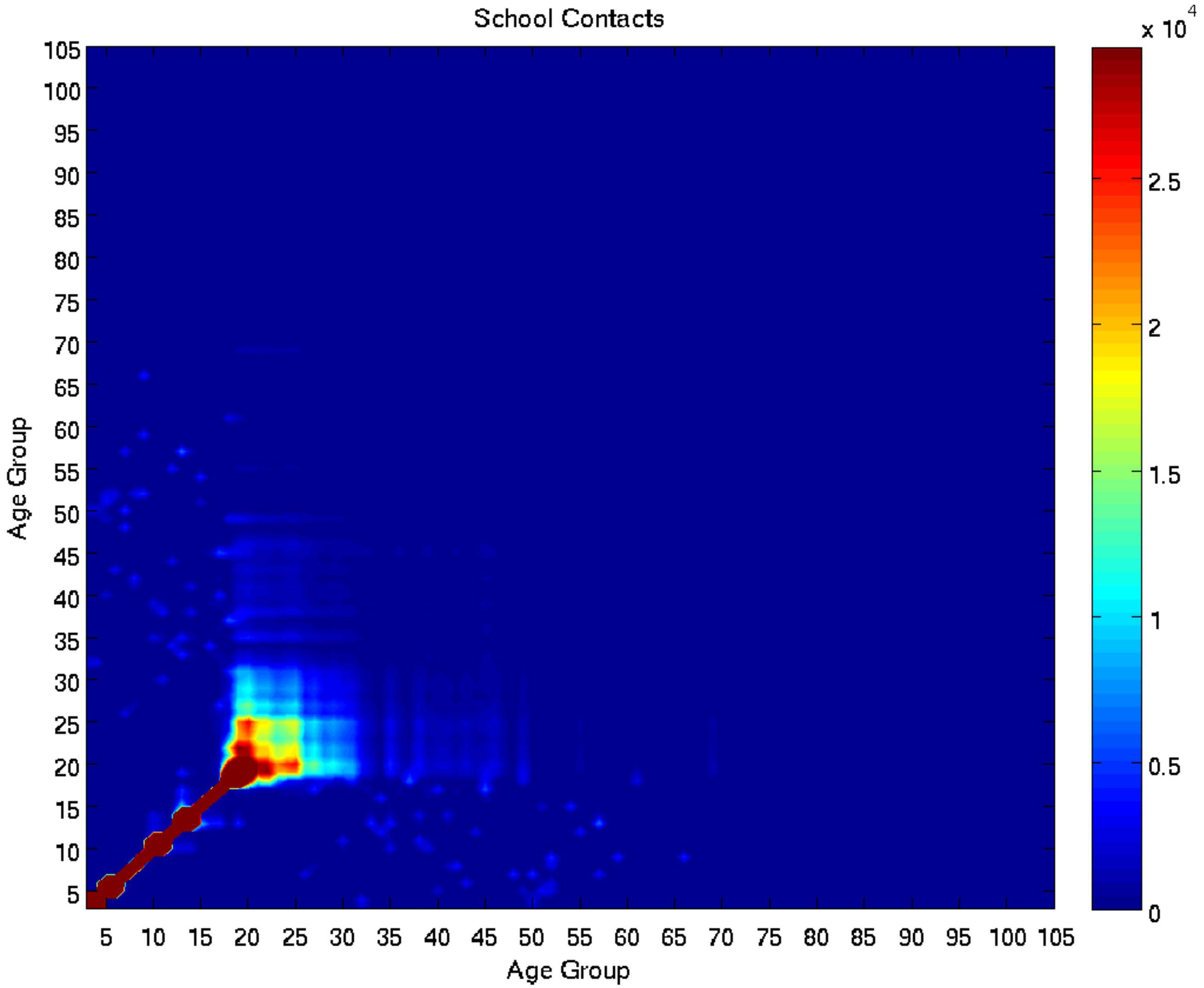
School Contacts
Age Group
Age Group
x 10^4
5
10
15
20
25
30
35
40
45
50
55
60
65
70
75
80
85
90
95
100
105
0
0.5
1
1.5
2
2.5

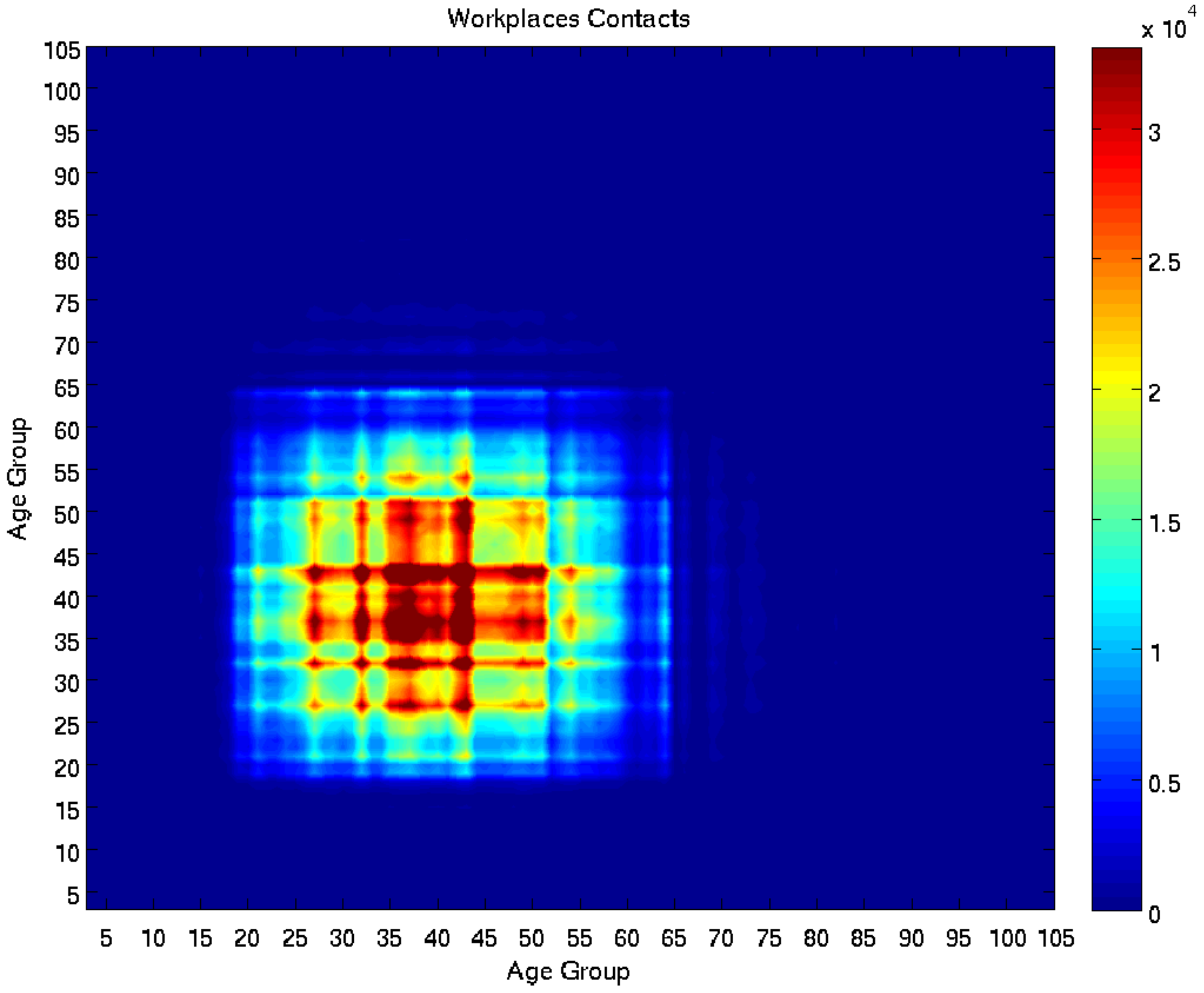

Workplaces Contacts
x 10^4
105
100
95
90
85
80
75
70
65
60
55
50
45
40
35
30
25
20
15
10
5
Age Group
5 10 15 20 25 30 35 40 45 50 55 60 65 70 75 80 85 90 95 100 105
Age Group
3
2.5
2
1.5
1
0.5
0

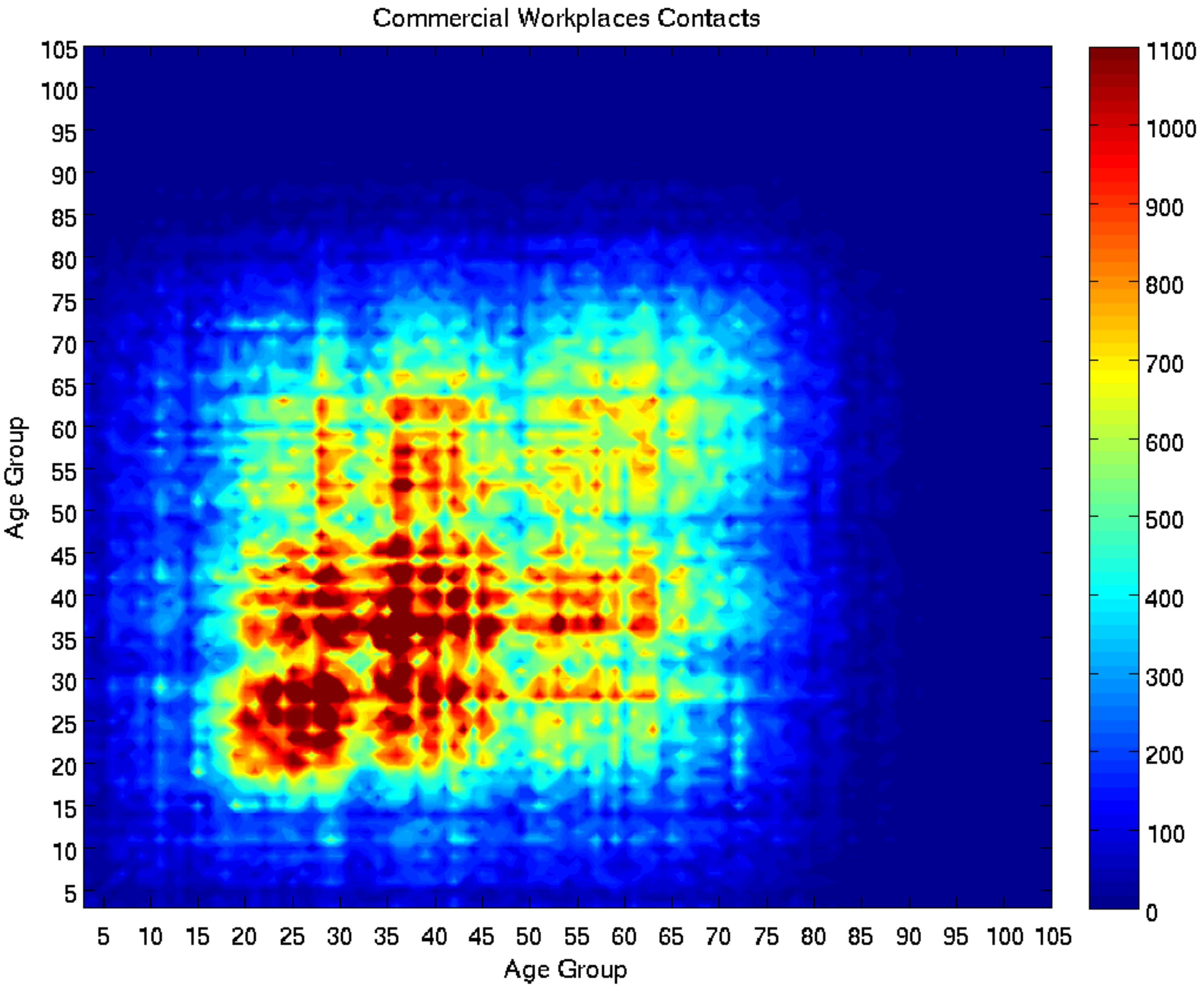
Commercial Workplaces Contacts
Age Group
Age Group
5 10 15 20 25 30 35 40 45 50 55 60 65 70 75 80 85 90 95 100 105
105 100 95 90 85 80 75 70 65 60 55 50 45 40 35 30 25 20 15 10 5
1100 1000 900 800 700 600 500 400 300 200 100 0

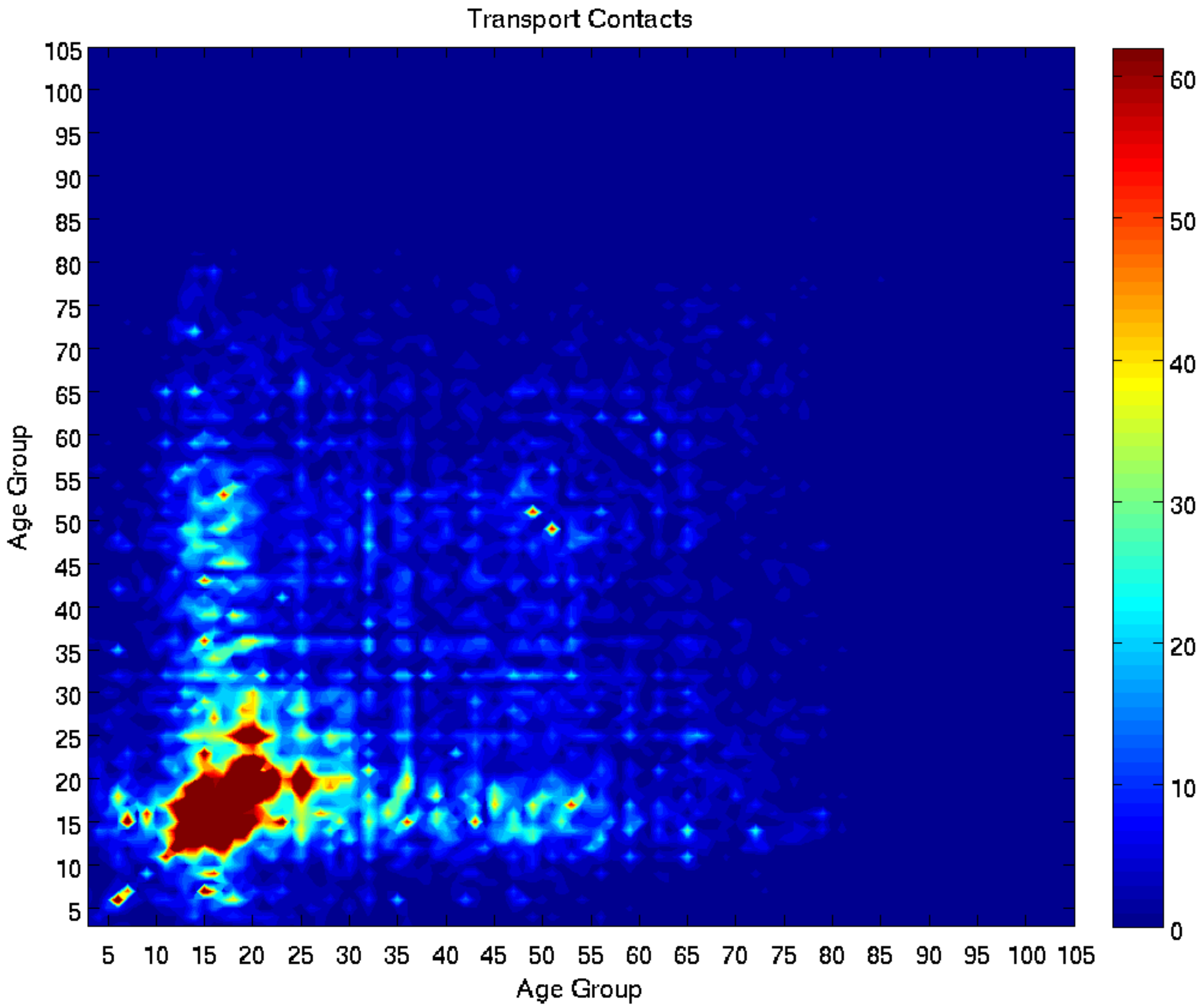
Transport Contacts
Age Group
Age Group
5
10
15
20
25
30
35
40
45
50
55
60
65
70
75
80
85
90
95
100
105
0
10
20
30
40
50
60
